# Immunocytochemical detection of *Mycobacterium Tuberculosis*complex specific antigen, MPT64, improves diagnosis of tuberculous lymphadenitis and tuberculous pleuritis

**DOI:** 10.1186/s12879-014-0585-1

**Published:** 2014-11-25

**Authors:** Agerie Tadele, Demissew Beyene, Jemal Hussein, Tuffa Gemechu, Asaye Birhanu, Tehmina Mustafa, Aster Tsegaye, Abraham Aseffa, Lisbet Sviland

**Affiliations:** University of Gondar, Gondar, Ethiopia; Addis Ababa University, Addis Ababa, Ethiopia; Armauer Hansen Research Institute, Addis Ababa, Ethiopia; Tikur Anbessa Specialized Hospital, Addis Ababa, Ethiopia; United Vision Medical Services, Addis Ababa, Ethiopia; Centre for International Health, Department of Global Public Health and Primary Care, University of Bergen, Bergen, Norway; Department of Thoracic Medicine, Haukeland University Hospital, Bergen, Norway; Department of Pathology, Haukeland University Hospital, Bergen, Norway; Gade Laboratory for Pathology, Department of Clinical Medicine, University of Bergen, Bergen, Norway

**Keywords:** Tuberculous lymphadenitis, Tuberculous pleural effusion, M. tuberculosis, ICC, MPT64 antigen

## Abstract

**Background:**

A rapid, sensitive and accurate laboratory diagnosis is of prime importance in suspected extrapulmonary tuberculosis (EPTB) cases. However, traditional techniques for the detection of acid-fast bacilli have limitations. The aim of the study was to evaluate the diagnostic value of immunocytochemical staining for detection of *Mycobacterium tuberculosis* complex specific antigen, MPT64, in aspirates from pleural effusions and lymph nodes, the most common presentations of EPTB.

**Method:**

A cross-sectional study was conducted by including patients at Tikur Anbessa Specialized Hospital and the United Vision Medical Services from December 2011 to June 2012. Lymph node aspirates and pleural fluid samples were collected and analyzed from a total of 51 cases (26 tuberculous (TB) pleuritis and 25 TB lymphadenitis) and 67 non-TB controls. Each specimen was subjected to Ziehl-Neelsen (ZN) staining, culture on Lowenstein– Jensen (LJ) medium, cytological examination, Polymerase Chain Reaction (PCR) using IS1081gene sequence as a primer and immunocytochemistry (ICC) with polyclonal anti-MPT64 antibody. All patients were screened for HIV.

**Result:**

ICC was positive in 38 of 51 cases and in the 7 of 67 controls giving an overall sensitivity and specificity of 74.5% and 89.5%, respectively. Using IS1081-PCR as a reference method, the sensitivity and specificity, positive and negative predictive value of ICC was 88.1%, 89.5%, 82.2% and 93.2%, respectively. The case detection rate increased from 13.7% by ZN stain to 19.6% by LJ culture, to 66.7% by cytology and 74.5% by ICC.

**Conclusion:**

Immunocytochemistry with anti-MPT64 antigen improved detection of TB in pleural effusion and lymph node aspirates. Further studies using monoclonal antibodies on samples from other sites of EPTB is recommended to validate this relatively simple diagnostic method for EPTB.

**Electronic supplementary material:**

The online version of this article (doi:10.1186/s12879-014-0585-1) contains supplementary material, which is available to authorized users.

## Background

Tuberculosis (TB) is an important public health problem in Ethiopia. According to World Health Organization (WHO) report of 2011, the estimated prevalence of TB cases in Ethiopia in 2010 was 394 per 100,000 and Ethiopia rank eighth among the 22 high TB- burden countries [1].

*Mycobacterium tuberculosis* (*M. tuberculosis*) has the ability to colonize almost any site in the body and it can affect organs other than the lung such as; pleura, lymph nodes, abdomen, genitourinary tract, skin, joints, bones and meninges [2],[3].

According to the TB and leprosy control program of Ethiopia, in the year 2010/2011, pulmonary TB accounts for 67.5% and extrapulmonary tuberculosis (EPTB) for 32.5% of all the new TB cases [4]. The most prevalent type of EPTB is tuberculous lymphadenitis (TBLN) [5],[6] with tuberculous pleuritis (TBP) being the second most frequent extrapulmonary manifestation [7],[8]. In spite of these high numbers, the diagnosis of EPTB remains a challenge for both clinicians and microbiologists [9],[10], because the disease presents in different ways and lack of diagnostic resources in developing countries adds to the problem. Diagnosis of EPTB is difficult due to its paucibacillary nature and the irregular distribution of bacilli that tend to clump together resulting in very low sensitivity of conventional acid fast bacilli (AFB) smear and culture [11],[12].

A recent study has examined the performance of an immunocytochemical (ICC) staining method for the detection of *M. tuberculosis* complex specific antigen; MPT64 in the diagnosis of EPTB. In this study ICC showed an overall sensitivity of 67.4% and a specificity of 95% [13]. MPT64 is a 26-kd secreted protein produced by *M. tuberculosis* complex organisms, and this antigen has not been detected in non-tuberculous mycobacteria. But, this antigen is absent in BCG strains with RD2 deletion [14].

The aim of our study was to evaluate the diagnostic ability of ICC staining to diagnose TBLN and TBP by the detection of *M. tuberculosis* complex specific antigen, MPT64 in a patient population from Ethiopia and compare the results with conventional methods i.e. Ziehl-Neelsen (ZN) staining and Lowenstein– Jensen (LJ) culture and polymerase chain reaction (PCR).

## Methods

### Study design, participants and specimens

Patients were included in the study from Tikur Anbessa Specialized Hospital and the United Vision Medical Services, Addis Ababa, Ethiopia from December 2011 to June 2012 by employing a cross-sectional study design. Pleural fluid specimens were collected consecutively from inpatients with a clinical diagnosis of TBP (n = 26), parapneumonic effusion (n = 14), pleural effusion secondary to malignancy (n = 7), cardiac failure (n = 6), cirrhosis (n = 5), chronic kidney disease (n = 5). Whereas lymph node aspirates were collected from ambulatory patients who were consecutively referred to United Vision Medical Services for fine needle aspiration cytology (FNAC) with a diagnosis of TBLN (n = 25), lymphadenopathy secondary to reactive condition other than TB (n = 19) and Hodgkin’s lymphoma (n = 11). Patients with active pulmonary TB on chest x-ray, patients receiving antituberculosis treatment prior to fine needle aspiration (FNA) or to thoracocentesis, and patients with contraindications to thoracocentesis were not included in the study.

A detailed clinical history (including sign and symptoms like, cough, fever and weight loss), physical examination, hemogram, tests for HIV, and chest X-ray were conducted for each study participant. The collected specimens were processed for ZN staining, culture on LJ medium and cytological examination. Deoxy ribonucleic acid (DNA) was extracted from pleural fluid and lymph node aspirates and PCR was performed using primers specific for IS1081. Immunocytochemical staining was performed with anti-MPT64 polyclonal antibody on smears from the sediment of pleural fluid and fine needle aspirate of the enlarged lymph node.

Patients were considered as cases when there was a clinical suspicion of TBLN or TBP and with a laboratory confirmation with either of the following tests, acid fast bacilli on ZN stain, and/or positive mycobacterial culture on LJ medium and/or positive by IS1081 based PCR and/or suggestive cytological finding of TB on Wright stained smear. Controls for the current study were patients with a clinical diagnosis other than TB and the absence of TB is confirmed by the above mentioned laboratory tests.

### Ethical consideration

Informed consent was obtained from each study participant and confidentiality was ensured. The decision to do a thoracocentesis or fine needle aspiration was made on clinical ground and not for the sake of participation in this study. Ethical approval was obtained from the departmental research ethical review committee of the department of Medical Laboratory Sciences, Addis Ababa University, Armauer Hansen Research Institute (AHRI) and All Africa Leprosy, Tuberculosis and Rehabilitation Training Centre (ALERT) Ethics Review Committee and the National Research Ethics Review Committee of Ethiopia.

### Fluids and aspirates

Consecutive pleural fluid samples (5-8 ml), aseptically aspirated, were collected in falcon tubes containing heparin (2 mg/ml) and sent to Microbiology department of Tikur Anbessa Specialized Hospital for microbiological analysis. Fine needle aspiration was performed from palpable lymph nodes of suspected cases by an experienced pathologist as an outpatient procedure using 21 Gauge needle attached to 10 ml syringe following standard procedure. One half of the fluid and aspirate sample was used for culture and DNA extraction. The other half was used for preparation of three smears; one was fixed for cytological examination, the second half for ZN stain and the remaining smear for ICC staining. The slide for ICC was fixed with absolute alcohol, covered with aluminum foil and stored at -20°C till processed.

### ZN stain and mycobacterial culture

Standard ZN staining procedure was applied with Clin-Tech Ziehl-Neelsen staining method kit (Clin-Tech Ltd, Unit G Perram Woks, United Kingdom). Three tubes of LJ medium (Fluka Analytical, Sigma-Aldrich, Switzerland) (two tubes with glycerol and one with sodium pyruvate) were used for inoculation and samples were incubated at 35 ± 2°C. The inoculated tubes were examined up to 8 weeks and colonies grown on LJ medium were confirmed by ZN staining.

To ensure the quality of ZN staining the following quality control measures were taken. A positive (*M. tuberculosis*) and a negative (mixed non acid fast organisms) control sputum smears were stained each time an acid fast stain was performed, if the controls did not show the proper reaction, patient and control sample were re-stained. Besides this, the entire positive and 10% of the negative slides were re-read by experienced laboratory technicians.

As a quality control measure for LJ culture, sterility and performance testing was done. Sterility testing was carried out by taking 3-5% of each batch and incubated at 35°C for 2 days, the rest was kept refrigerated. If more than two colonies per tube are seen, the whole batch of the LJ media was discarded. Performance testing for LJ media was done by streaking the stock strains of *Mycobacterium gordonae* (fast grower mycobacterium) on LJ tube. Overnight incubation was then done at 37°C and the good quality of LJ medium was assured by growth seen after an overnight incubation.

### Cytological examination

Cytological examination of lymph node aspirates and pleural fluids was done by staining with Wright solution and the examination was done by an experienced pathologist. Aspirates from lymph nodes were diagnosed as TBLN based on the presence of granulomas consisting of epithelioid cells/giant cells without caseation necrosis (Group I), necrotizing granulomas having variable degree of necrosis with scattered well-formed granulomas (Group II) and largely necrotic material with neutrophils, lymphocyte and a few scattered epithelioid cells (Group III).

TBP was diagnosed by cytological findings of lymphocytic pleural effusion in a proteinaceous or fluidy background, devoid of neoplastic cells.

### Polymerase chain reaction assay

#### DNA extraction from lymph node aspirate and pleural fluid

Genomic DNA extraction was performed as previously described by Arora et al. and Pahwa et al. [15],[16]. Briefly, 400 μl of the FNA material was incubated in water bath at 80°C for 30 minutes and then diluted with 500 μl of Tris-EDTA (TE) buffer (10 mM Tris, 1 mM EDTA, pH 8.0). Similarly, 400 μl of pellet from a centrifuged pleural fluid sample was resuspended in 500 μl of TE buffer. The bacteria were then lysed with 50 μl lysozyme (10 mg/ml) (Sigma-Aldrich, Chemie Gmbh, Steinheim, Germany) and incubated for 1 hour at 37°C for lymph node aspirate and 2 hours for pleural fluid.

The lysozyme-treated samples were incubated at 65°C for 10 minutes and 1 hour in the presence of 5 and 6 μl of 10 mg/ml of proteinase K (BDH Biochemical, VWR International LTD, England) for lymph node and pleural fluid sample respectively in the presence of 70 μl of 10% sodium dodecylsulphate (SDS). A 100 μl of 5 M solution of sodium chloride and subsequently a 100 μl pre-warmed cetyltrimethyl ammonium bromide (CTAB)/sodium chloride solution were added before incubating in water bath at 65°C for 10 minutes. Then phenol/chloroform/isoamyl alcohol (25:24:1) extraction was performed. The DNA precipitate was obtained by adding absolute alcohol after overnight incubation at -20°C and the DNA was pelleted by centrifugation at 12,000 rpm for 15 minutes, then washed with cold 70% ethanol, and resuspended in 30 μl of TE buffer. The concentration of the extracted DNA was approximated by running it on a 1% agarose gel electrophoresis. Finally, the DNA samples were stored at -20°C until used for PCR.

The quality of the DNA extraction protocol was first assured by running known samples of pleural fluid and lymph node aspirate which contain mycobacterial DNA. A positive sample and a negative control, i.e. TE buffer in place of sample were run in each batch of DNA extraction.

### Polymerase chain reaction

PCR was performed using IS-1081 (an insertion sequence element which is found in multiple copies of five to seven) within the chromosome of all strains belonging to *M. tuberculosis* complex) primers (forward and reverse), Hot StartTaq Master Mix (Qiagen, Germantown, MD, USA) and QiagenRNase-Free Water (Germantown, MD, USA). A total of 25 μl, PCR reaction mix containing 4 μl of the extracted DNA, 10 μl of HotStarTaq master mix, 0.5 μl of each forward and reverse primers and 5 μl of RNase-Free water was used for PCR amplification. The PCR amplification was carried out for 35 cycles, where each cycle was 95°C for 1 minute, 55°C for 30 seconds, and 72°C for 30 seconds with an initial heat activation step of 95°C for 15 minutes and a final extension of 72°C for 10 minutes using TC 512 Thermocycler (Barworld Scientific, England). The amplified product was analyzed in a 2% agarose gel containing ethidium bromide (Fisher Bioreagents, Fair Lawn, USA). Positive samples showed a band at 136 base pair and pictures of the gels were taken after exposing to UV light for documentation.

PCR-positive and PCR-negative controls were processed in all PCR assays. Three types of PCR positive controls were included: (i) *M. tuberculosis* strain H37Rv DNA, (ii) known ZN positive and culture-positive test sample and (iii) positive sample from previous PCR run. Similarly, three types of PCR-negative controls were also processed in each set of assays: (i) an extraction control (with all the steps but without any extracted material), (ii) a reaction tube with substitution of Qiagen RNase free-water for the test template (iii) a negative sample from previous PCR assay. To check for cross-contamination, random samples were retested. Separate laboratory spaces which were dedicated to the process of DNA extraction, PCR mixture preparation, cycling and electrophoresis were used.

### Immunocytochemical staining

**Figure 1:**
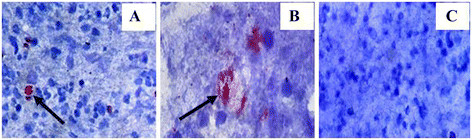
**Immunocytochemical staining of lymph node aspirates (A), pleural fluid (B) and negative lymph node aspirate sample (C).** Positive staining is seen as reddish brown within cells and extracellular in necrotic areas as indicated by the arrow in A and B. Pattern of the stained positive cells may be granular or diffuse.

Immunocytochemical staining was performed according to Purohit et al. [13]. Briefly, ICC staining was done with DakoCytomation kit (EnVision + System-HRP; DakoCytomation Denmark A/S, Glostrup, Denmark) on all smeared fluid and aspirate samples to demonstrate the presence of MPT64 *M. tuberculosis* complex specific antigen. The alcohol-fixed smears were hydrated through decreasing grades of alcohol, washed in phosphate buffered saline (PBS) for 10 min, and treated with 3% H_2_O_2_ for 15 minutes and then incubated with in-house absorbed polyclonal anti-MPT64 antibody (received from the University of Bergen, Norway) at 1/250 dilution. Subsequent incubations were then performed with anti-rabbit dextran polymer conjugated to horseradish peroxidase and with the substrate consisting of 3-amino-9-ethylcarbazol (AEC) and hydrogen peroxide to visualize bound antibodies. Finally, the background was counterstained with Mayer’s hematoxylin, and the slides were mounted in Dako Faramount aqueous immunomount for microscopy. All incubations were carried out at room temperature, and the slides were washed for 3 minutes three times with 0.05 M tris-buffered saline-tween 20 between incubations. Mycobacterium antigen was stained reddish brown within the cytoplasm of macrophages or extracellularly in necrotic materials. Pattern of the intracellular staining might be granular or diffuse (Figure 1).

In each staining batch, one positive control and two negative controls were included. The positive control contains known sample containing *M. tuberculosis*. In one negative control, the primary antibody was substituted with antibody diluent and in the other negative control an irrelevant rabbit polyclonal antibody (anti- human chorionogonadotrophin) was used to evaluate the specificity of the reaction.

### Statistical analysis

Double entry of data was done using Microsoft Excel 2007 and analysis was done using SPSS Version 20. GraphPad Prism 5 was also used to construct figures. The performance indices (the sensitivity, specificity, positive and negative predictive values, false positive and false negative rates) of all the modalities was calculated and compared between cases and controls. The performance indices are calculated using combination of tests as well as IS1081- PCR as a reference method. Pearsons Chi-square test and Fisher’s exact tests were used to compare differences of categorical variables and the significance was set at a P value of <0.05.

## Results

A total of 118 samples (63 pleural fluid and 55 lymph node aspirates) were collected from patients attending Tikur Anbessa Specialized Hospital and United Vision Medical Service from December 2011 to June 2012. Of the total study participants, 51 were TB cases (26 cases of TBP and 25 cases of TBLN) and 67 were controls (37 were pleural fluid and 30 were lymph node aspirates).

### Clinical data

In our study, participants from all age groups were included ranging from 3 to 85 years with a median age of 35 years. Majority [52/118; (44.1%)] of the study participants were between 25 to 44 years. Male study participants constitute 53.4% (63/118) of the total sample and females constitutes 46.6%. Twenty seven point five percent (14/51) of TB cases and 19.4% (13/67) of non-TB controls were HIV seropositive.

All patients presented with signs and symptoms according to the site of the diseases. The predominating local symptom for TBP cases was cough, chest pain, loss of weight and night sweating. While, the predominating local symptom in study participants with TBLN was neck mass, night sweating, loss of appetite and headache (Table 1). From a total of 55 participants with lymphadenopathy, the majority 43 (78.2%) of them had cervical lymph node swelling followed by axillary 7 (12.7%) and submandibular 5 (9.1%) lymph nodes.

**Table 1:** **Signs and symptoms of study participants by type of extrapulmonary pulmonary tuberculosis**

Sign and symptom	Pleural fluid (N = 63)		Lymph node aspirate (N = 55)	
	Cases (n = 26)	Controls (n = 37)	Cases (n = 25)	Controls (n = 30)
Neck mass	0(0%)	0(0%)	23(92.0%)	28(93.3%)
Headache	16(61.5%)	6(16.2%)	18(72.0%)	26(86.7%)
Chest pain	23(88.5%)	21(56.8%)	4(16.0%)	2(6.7%)
Cough	24(92.3%)	23(62.2%)	6(24.0%)	3(10.0%)
Fever	17(65.4%)	16(43.2%)	15(60.0%)	9(30.0%)
Tiredness	6(23.1%)	14(37.8%)	15(60.0%)	19(63.6%)
Loss of weight	21(80.8%)	23(62.2%)	16(64.0%)	23(76.7%)
Loss of appetite	23(88.5%)	18(48.6%)	18(72.0%)	17(56.7%)
Night sweating	19(73.1%)	13(35.1%)	18(72.0%)	12(40.0%)

### Microbiological analysis

As shown in Table 2, ZN staining was positive in 7/51 (13.7%) TB cases and 10/51 (19.6%) of the samples demonstrated growth on LJ media. The bacilli load of the positive pleural fluid sample was 3 bacilli/100 fields. Unlike that of pleural fluid samples, AFB was seen in 6/26 (24%) of lymph node aspirates from cases of TBLN. Out of the 6 ZN staining positive lymph node aspirates, 1+, 2+ and 4 bacilli/100 fields was seen in 3(50%), 2(33.3%) and 1(16.7%) lymph node aspirates respectively as graded by WHO/International Union against Tuberculosis and Lung Disease (WHO/IUATLD). Among the 10 culture positive samples, 5 (50%) grew on glycerol containing media, 3(30%) on both glycerol and sodium pyruvate containing media and the remaining 2 (20%) grew on sodium pyruvate.

**Table 2:** **Laboratory results of different diagnostic procedures in tuberculous pleuritis and tuberculous lymphadenitis cases and non-TB controls**

Diagnosis	ZN stain	Number of specimens n(%) positive by			
		LJ culture	Cytology	IS1081-PCR	ICC
TB (51)					
Pleural fluid (n = 26)	1(3.8)	3(11.0)	16(61.5)	21(80.8)	18(69.2)
Lymph node aspirate (n = 25)	6(24.0)	7(28.0)	18(72.0)	21(84.0)	20(80.0)
Total	7(13.7)	10(1.6)	34(66.7)	42(82.4)	38(74.5)
Non TB (N = 67)					
Pleural fluid (n = 37)	0(0.0)	0(0.0)	0(0.0)	0(0.0)	5(13.5)
Lymph node aspirate (n = 30)	0(0.0)	0(0.0)	0(0.0)	0(0.0)	2(6.7)
Total	0(0.0)	0(0.0)	0(0.0)	0(0.0)	7(10.4)

### Cytological examination

Cytological examination of pleural fluids and lymph node aspirates revealed that 16/26 (61.5%) and 18/25 (72.0%) of TBP and TBLN cases were diagnosed as suggestive of TB respectively (Table 2). Forty four percent (11/25) of TBLN smears showed group I cytomorphologic change, while group II and III cytomorphologic change was observed in 8/25 (32%) and 6/25 (24%) of the lymph node aspirates. As it is described in Table 3, higher percentage of positivity of AFB, LJ culture, PCR and ICC was observed in group II (granuloma with necrosis) cytomorphologic pattern compared to the others.

**Table 3:** **The positivity of different laboratory tests among various cytomorphologic patterns from lymph node aspirates of cases with tuberculous lymphadenitis**

	Cytomorphologic pattern (n = 25)			
	Non necrotic granuloma (n = 11) n (%)	Necrotic granuloma (n = 8) n (%)	Necrotic material	
			(n = 6)	n (%)
Laboratory tests				
**AFB**	1(9)	3(38)	2(33)	
**LJ culture**	2(18)	4(50)	1(17)	
**PCR**	9(82)	7(88)	5(83)	
**ICC**	9(82%)	6(75)	5(83)	

### Polymerase chain reaction

**Figure 2:**
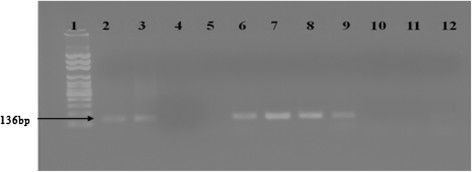
**IS1081-PCR of DNA extracted directly from pleural fluid and lymph node aspirate of lane 1 represents a 100 bp DNA ladder (molecular marker), Lane 2 through lane 4 are DNA extracted from pleural fluid, Lane 5 and lane 6 are DNA extracted samples from lymph node aspirates, Lane 7 thorough 9 represent an amplification product of the positive controls, i.e.** ***M. tuberculosis*** **strain H37Rv DNA, known ZN-positive and culture-positive test sample, and positive sample from previous PCR run which showed positive result, Lane 10 through 12, i.e. an extraction control, a reaction with substitution of Qiagen RNase free-water for the test template, and a negative sample from previous PCR assay gave a negative result.**

Out of 51cases of TB, 42 (82.4%) of the study participants were positive by IS1081-PCR. Among cases with TBP and TBLN, 21 (80.8%) and 21 (84.0%) were positive by IS1081-PCR respectively (Table 2). Figure 2 depicts a representative gel showing a positive (136 bp) and negative pleural fluid and lymph node aspirate.

### Immunocytochemical staining

Of 51 cases of tuberculous pleuritis and tuberculous lymphadenitis, ICC staining with anti-MPT64 was positive in 38 of smears and 7 controls, giving the overall sensitivity, specificity, positive predictive value and negative predictive value of 74.5, 89.5, 84.4 and 82.2%, respectively. All ZN and culture positive tuberculous smears were positive with ICC using anti-MPT64 antibody.

### Comparison of diagnostic procedure among cases

**Figure 3:**
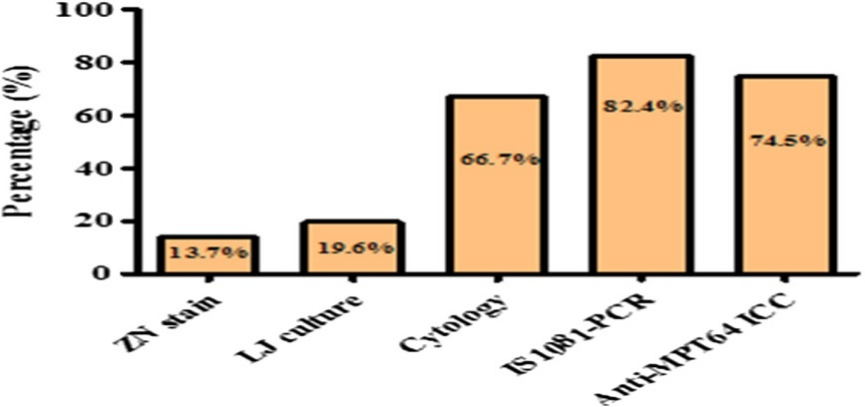
**Positivity of laboratory tests among cases.**

The positivity of ZN, LJ culture, cytologic examination, IS1081-PCR and ICC staining with anti-MPT64 antibody among 51 cases of TBP and TBLN, as defined by the combination of tests, was compared. As it is shown in Figure 3, the diagnosis of TB has increased from 7 (13.7%) samples by ZN stain to 10 (19.6%) by LJ culture. The case detection further increased to 34 (66.7%) by cytological examination and to 42 (82.4%) with IS1081-PCR. ICC alone detected 38 (74.5%) cases. From Figure 3, it is clear that ICC could detect significantly more cases of TB when compared to the conventional AFB, culture and cytological examination.

### Diagnostic evaluation of immunocytochemistry

ICC was positive among 38 tuberculous cases and 7 controls, giving an overall sensitivity and specificity of 74.5% and 89.5%, respectively. Diagnostic validity of ICC was performed using PCR as a reference method. The sensitivity, specificity, positive and negative predictive value of ICC among cases was 88.1%, 89.5%, 82.2% and 93.2%, respectively. The diagnostic parameters are higher among cases with TBLN than TBP (Table 4).

**Table 4:** **Diagnostic evaluation of immunocytochemistry for pleural fluid and lymph node aspirate**

Type of EPTB	Sensitivity (%)	Specificity (%)	PPV (%)	NPV (%)
TBP	81.0	88.2	68	93.5
TBLN	95.2	90.1	74.1	98.5
Total	88.1	89.5	82.2	93.2

### Positivity of different laboratory tests by HIV serostatus

There was no statistically significant difference with the positivity of ZN staining, LJ culture, cytology and IS1081-PCR tests among participants with reactive and non reactive HIV serostatus (Table 5). But, a statistically significant higher number of HIV negative cases were positive with immunocytochemical staining as compared to the HIV positive cases (p-value = 0.013).

**Table 5:** **Positivity of laboratory tests among HIV reactive and non-reactive study participants**

Laboratory tests	HIV Seros taus		
	Reactive n (%)	Non reactive n (%)	P-value
ZN staining	2(28.6)	5(71.4)	0.659
LJ Culture	2(20.0)	8(80.0)	0.707
Cytology	11(32.4)	23(67.6)	0.148
IS1081-PCR	12(28.6)	30(71.4)	0.360
ICC	16(35.6)	29(64.4)	0.013

## Discussion

The present study aimed to investigate the diagnostic value of ICC to detect *M. tuberculosis* specific antigen, MPT64, for the diagnosis of the most common presentations of EPTB, TBLN and TBP in pleural fluid and lymph node aspirate samples from Tikur Anbessa Specialized Hospital and United Vision Medical Services, Addis Ababa, Ethiopia. The results showed that ICC using MPT64 antigen is a sensitive and specific test for diagnosis of TBLN and TBP. This study confirms the previous study done on patients from India showing that ICC using polyclonal anti-MPT64 antibody can be used to achieve a rapid and early diagnosis of TBLN, TBP and TB meningitis [13].

The results of this study indicated that only 1/26 (3.8%) of pleural fluid sample from study participants with TBP was positive for AFB and the bacillary load of the positive smear was 3 bacilli/100 microscopic fields. This finding is in line with other studies, which demonstrated no bacilli [13],[17]-[19] or found lower detection rates like 0.9% positivity rate of AFB in Myanmar [20], and 3.8% positivity rate of AFB was observed in other study [21]. The low positivity of pleural fluid for ZN staining is attributed to the pathophysiology of tuberculous pleural effusion, in that the basic mechanism of TBP is immunologic, i.e. fluid collects as a result of a delayed hypersensitivity reaction to tuberculous proteins [22].

Unlike that of the pleural fluid sample, 24% of cases of TBLN were positive for AFB and a similar percentage of AFB positivity was observed in a study by Aljafari et al. [23]. The positivity of pleural fluid on LJ culture was 11.5% and this finding is in agreement with other studies, 15.4% [19], 19% [13]. This may reflect the paucibacillary status of pleural fluid which results at least partly from immunologic mechanism. The other possible reason for low sensitivity of LJ culture on pleural fluid might be associated with the small volume of sample. Besides its low sensitivity, mycobacterial culture requires biosafety level 3 laboratories challenging the implementation in resource poor settings. However, the possibility of drug sensitivity testing by culture makes it an important and irreplaceable diagnostic tool. Lack of this possibility by ICC is a weakness of this method.

The positivity rate of culture among TBLN cases in our study was 28% and is comparable with other studies which revealed 24.4% [24] LJ culture positivity of lymph node aspirates. But it was lower than the sensitivity of LJ culture of lymph node aspirates in another study which has reported a culture positivity of 38.8% [25]. The low sensitivity in our study might be explained by stage of the lymphadenitis, where high number of tubercle bacilli is present in central abscess/ necrotic material [26].

Among cases with TBLN, a higher positivity of AFB, LJ culture, IS1081-PCR was observed in group II (necrotizing granulomas) cytomorphologic pattern and this finding was consistent with other studies showing higher degree (75.6%) of positivity of AFB [27], culture (26%) among cases with cytomorphological pattern of necrotizing granulomas [13]. But, a slightly higher positivity was observed with ICC in cases with non necrotizing cytomorphologic pattern (Table 3), which is consistent with the previous study by Purohit et al. This implies that ICC has a great role in the diagnosis of the most difficult cytomorphologic pattern.

IS1081-PCR was positive among 42/51 (82.4%) cases of TBP and TBLN and it was positive in 21/26 (80.8%) of TBP cases and this result agrees with a study conducted to evaluate the performance of IS1081on TBP which showed a sensitivity of 84.6% [21]. IS1081-PCR had increased the sensitivity of detection of cases of TB both in TBP and TBLN. As it was depicted in Figure 3, IS1081-PCR has increased the case detection of TB in TBP and TBLN in comparison to LJ culture. PCR positivity of several cases that were culture negative indicates that PCR is very sensitive and useful in picking up cases that may harbor dead bacilli [16].

Using PCR as the gold standard and the sensitivity, specificity, positive predictive value and negative predictive value were 88.1, 89.5, 82.2 and 93.2%, respectively. This finding was lower compared to a similar study which was conducted in India in 2006, where the reported sensitivity and specificity, positive predictive value and negative predictive value were 96, 96, 95 and 97%, respectively. The difference in performance characteristics may be explained by the variation in case selection criteria, type of extrapulmonary samples, the cytomorphologic pattern of lymph node aspirates, and the primer used for PCR amplification. As it is illustrated in Table 4, the sensitivity, specificity, positive predictive value and negative predictive value of the pleural fluid sample is lower than the lymph node aspirates. This might be due to the paucibacillary status of pleural fluid which results from immunologic mechanism. In this study, pre-treatment of pleural fluid sample for disruption of the immune complexes, in the view of antigen retrieval, was not done. This was based on the experience of the earlier study where pre-treatment (e.g. microwave treatment) did not improve the sensitivity [13]. This may be due to alcohol fixation of these samples which does not lead to cross-linking in a manner which happens with formalin-fixation of the biopsies, making antigen retrieval necessary.

There were 7 positive samples by ICC among the non-TB groups. The possible reason for having a false positive result might be non-specific staining due to the polyclonal nature of the antibody used, while the false negative might be due to sample volume [28], to the degradation of antigens with the length of time in storage [29], and the absence of MPT64 from some TB strains. Therefore the decision to treat a patient must always be made after a total evaluation based on the clinical suspicion, and the results of other diagnostic tests. Furthermore, study using a monoclonal anti-MPT64 antibody is recommended. The increase in TB case detection using ICC observed in this study may be attributed to the ability of ICC to detect fragments of antigen rather than the presence of the intact bacilli which is a prerequisite for ZN staining [30],[31]. The lower yields of ICC staining in pleural fluid as compared to lymph node aspirate could be due to presence of fewer bacilli at this site, as supported by lower positivity with culture. This lower yield of conventional and ICC method might also be explained by lack of adequate sample volumes due to apportioning of sample for various diagnostic tests [28].

## Conclusion

Immunocytochemical staining method showed an increased sensitivity in the diagnosis of TBP and TBLN compared with other conventional methods, such as ZN staining, culture and cytological examination. The use of ICC for the detection of mycobacterial complex specific antigen for clinical management should always be interpreted together with clinical history, examination, and other diagnostic tests since it does have false positive results. Furthermore, ICC needs to be evaluated by the monoclonal anti-MPT64 antibody, with larger number of sample from other types of EPTB. Otherwise, it is a simple method which does not require a sophisticated instrumentation, i.e. it requires an ordinary light microscope for examination of the stained preparations. It takes 3 and half hours, where patients can have the result within same day of collection. Due to its simplicity, ICC can be performed in a routine pathology laboratory.

## Authors’ contributions

AT: Designed the study, performed data collection, laboratory analysis, statistical analysis, interpreted the data and prepared first draft of the manuscript, DB: participated in designing the study, study site selection, optimization and close follow up on laboratory analysis, data analysis, interpreted data and manuscript write up, JH: participated in designing the study, cytological examination of the specimens; manuscript write up, TG: participated in patient recruitment and specimen collection, assisted in preparation of the manuscript, AB: assisted in designing the study and preparation of the manuscript, TM: assisted in designing study and statistical analysis, interpretation of data, preparation of manuscript, AsT: assisted in designing the study, participated in data analysis, interpretation of data and manuscript write up, AA: participated in designing the study, selection of study site, facilitated logistics, data analysis, interpretation of data and participated in preparation of manuscript, LS: participated in designing the study, facilitated logistics, data analysis, interpretation of data and manuscript preparation. All authors read and approved the final manuscript.

## Authors’ information

AT: MSc, Lecturer, University of Gondar, Gondar, Ethiopia.

DB: PhD, Post doctoral Scientists, Armauer Hansen Research Institute, Addis Ababa, Ethiopia JH: MD+, Pathologist, Armauer Hansen Research Institute, Addis Ababa, Ethiopia.

TG: MD+, Pathologist, TikurAnbessa Specialized Hospital, Addis Ababa, Ethiopia AB: MSc, Lecturer, Addis Ababa University, Addis Ababa, Ethiopia.

TM: PhD, Professor, Center for International Health, University of Bergen, Bergen, Norway AsT: PhD, Lecturer, Addis Ababa University, Addis Ababa, Ethiopia.

AA: PhD, Scientific Director, Armauer Hansen Research Institute, Addis Ababa, Ethiopia.

LS: PhD, Professor, Department of Pathology, Haukeland University Hospital Gade Laboratory for Pathology, Department of Clinical Medicine, University of Bergen, Bergen, Norway.

## Authors’ original submitted files for images

Below are the links to the authors’ original submitted files for images. Authors’ original file for figure 1 Authors’ original file for figure 2 Authors’ original file for figure 3

## Abbreviations

ICC: Immunocytochemistry
TBLN: Tuberculous lymphadenitis
TBP: Tuberculous pleural effusion
TB: Tuberculosis
PCR: Polymerase chain reaction
AFB: Acid fast bacilli
ZN: Ziehl-Neelsen
LJ: Lowenstein-Jensen
EPTB: Extrapulmonary tuberculosis

## References

[1] World Health Organization: *Global Tuberculosis Control WHO Report 2011.* Geneva: 2011.

[2] Cavalcante S, Chakaya JM, Egwaga SM, Gie R, Gondrie P, Harries AD: *Treatment of Tuberculosis: Guidelines* 2010:23-28.

[3] Gillespie SH (2006). Mycobacterium Tuberculosis. Principle and Practice of Clinical Bacteriology.

[4] Guidelines for Clinical and Programmatic Management of TB, TB/HIV and Leprosy in Ethiopia. 2013, 5

[5] Zenebe Y, Anagaw B, Tesfay W, Debebe T, Gelaw B: Smear positive extra pulmonary tuberculosis disease at University of Gondar Hospital Northwest Ethiopia. *BMC Research Notes* 2013, 6:21., 10.1186/1756-0500-6-21 PMC3558382 23331864

[6] Deribew A, Negussu N, Melaku Z, Deribe K: Investigation outcomes of tuberculosis suspects in the health centers of Addis Ababa Ethiopia. *Eth Med J* 2011, 6:e18614., 10.1371/journal.pone.0018614 PMC3079716 21526179

[7] Valdes L, Pose A, Jose ES, Vazquez JM (2003). Tuberculous pleural effusions. Eur J Intern Med.

[8] Engel M, Matchaba P, Volmink J: Corticosteroids for tuberculous pleurisy (Review). *The Cochrane Library* 2009:1-6., 10.1002/14651858.CD001876.pub2 17943759

[9] Hesselink DA, Yoo S-M, Verhoeven G, Brouwers JW, Smit FJ, van Saase JLCM (2003). A high prevalence of culture-positive extrapulmonary tuberculosis in a large Dutch teaching Hospital. Neth J Me.

[10] Golden MP, Vikram H (2005). Extrapulmonarytuberculosis: an overview. Am Fam Physician.

[11] Perkins M, Roscigno G, Zumla A (2006). Progress towards improved tuberculosis diagnostics for developing countries. Lancet.

[12] Boehme C, Bossuyt P, Campos-Neto A, Cuevas L, Dacombe R, Doherty M, Guillerm M, Hanson C, Jefferson C, Klaster P, Kolk A, Alexander H, Laal S, Mann G, Maher D, McNerney R, Menzies D, Moore D, O’Brien R, Pai M, Palomino JC, Perkins M, Portaels F, Ramsay A, Riguots L, Roscigno G, Salfinger M, Shinnick TM, Angerer TVS, Sohn H (2009). Pathways to better diagnostics for tuberculosis: a blue print for the development of tb diagnostics by new diagnostics working group of the stop tb partnership.

[13] Purohit MR, Mustafa T, Wiker HG, Sviland L (2012). Rapid diagnosis of tuberculosis in aspirate, effusions, and cerebrospinal fluid by immunocytochemical detection of *Mycobacterium tuberculosis*complex specific antigen MPT64. Diagn Cytopathol.

[14] Nagai S, Wiker HG, Harboe M, Kinomoto M (1991). Isolation and partial characterization of major protein antigens in the culture fluid of *Mycobacterium tuberculosis*. Infect Immun.

[15] Arora SK, Kumar B, Sehgal S (2000). Development of a polymerase chain reaction dot-blotting system for detecting cutaneous tuberculosis. Br J Dermatol.

[16] Pahwa R, Hedau S, Jain S, Jain N, Arora VM, Kumar N, Das BC (2005). Assessment of possible tuberculous lymphadenopathy by PCR compared to non-molecular methods. J Med Microbiol.

[17] Wongtim S, Silachamroon U, Ruxrungtham K, Udompanich V, Limthongkul S, Charoenlap P, Nuchprayoon C (1999). Interferon gamma for diagnosing tuberculous pleural effusions. Thorax.

[18] Hasaneen NA, Zaki ME, Shalaby HM, El-Mors AS (2003). Polymerase chain reaction of pleural biopsy is a rapid and sensitive method for the diagnosis of tuberculous pleural effusion. Chest.

[19] Salleh SA, Hussin S, Rahman MM (2008). Nested PCR for rapid detection of TB from Pleural fluid at HUKM Malaysia. Pak J Biol Sci.

[20] Soe Z, Shwe WH, Moe S (2010). A **study on tuberculous pleural effusion**. IJCRIMPH.

[21] Bahador A, Etemadi H, Kazemi B, Ghorbanzadeh R, Nakhjavan FA, Nejad ZA (2005). Performance assessment of IS1081-PCR for direct detection of tuberculous pleural effusion: compared to rpoB-PCR. Res J Agric and Biol Sci.

[22] Valdes L, Alvarez D, Jose ES, Penela P, Valle JM, Garcia-Pazos JM, Suárez J, Pose A (1998). Tuberculous pleurisy: a study of 254 patients. Arch Intern Med.

[23] Aljafari AS, Khalil EAG, Elsiddig KE, Hag IAE, Ibrahim ME, Elsafi ME, Hussein AM, Elkhidir IM, Sulaiman GS, Elhassan AM (2004). Diagnosis of tuberculous lymphadenitis by FNAC, microbiological methods and PCR: a comparative study. Cytopathol.

[24] Purohit MR, Mustafa T, Sviland L (2008). Detection of *Mycobacterium tuberculosis*by polymerase chain reaction with DNA eluted from aspirate smears of tuberculous lymphadenitis. Diagn Mol Pathol.

[25] Derese Y, Hailu E, Assefa T, Bekele Y, Mihret A, Aseffa A, Hussien J, Ali I, Abebe M (2012). Comparison of PCR with standard culture of fine needle aspiration samples in the diagnosis of tuberculosis lymphadenitis. J Infect Dev Ctries.

[26] Suh KW, Park CS, Lee JT, Lee KG (1993). Diagnosis of cervical lymphadenitis with fine needle aspiration biopsy and cytologic examination under ultrasonographic guides. Yonsel Medical J.

[27] Ergete W, Bekele A (2000). Acid fast bacilli in aspiration smears from tubercuous patients. Ethiop J Health Dev.

[28] Chakravorty S, Sen MK, Tyagi JS (2005). Diagnosis of extrapulmonary tuberculosis by smear, culture, and PCR using universal sample processing technology. J Clin Microbiol.

[29] Haines DM, Chelack BJ (1991). Technical considerations for developing enzyme immunohistochemical staining procedures on formalin-fixed paraffin-embedded tissuesfor diagnostic pathology. J Vet Diagn Invest.

[30] Ulrichs T, Lefmann M, Reich M (2005). Modified immunohistological staining allows detection of Ziehl-Neelsen-negative *Mycobacterium tuberculosis*organisms and their precise localization in human tissue. J Pathol.

[31] Gutierrez C, Garcia MJF (1993). Comparison of Ziehl-Neelsen staining and immunohistochemistry for detection of *Mycobacterium bovis*in bovine and caprine tuberculosis lesions. J Comp Pathol.

