# Supplementary figures and images for: Immunocytochemical detection of Mycobacterium Tuberculosiscomplex specific antigen, MPT64, improves diagnosis of tuberculous lymphadenitis and tuberculous pleuritis
Source: BMC Infect Dis. 2014 Nov 25;14:585. doi: 10.1186/s12879-014-0585-1 (PMC4262190; doi:10.1186/s12879-014-0585-1)

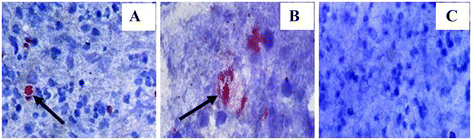

Supplement: Supplementary file 1 — Authors’ original file for figure 1 [file 12879_2014_585_MOESM1_ESM.gif]

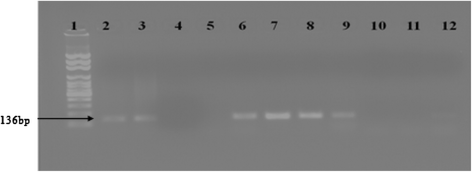

Supplement: Supplementary file 2 — Authors’ original file for figure 2 [file 12879_2014_585_MOESM2_ESM.gif]

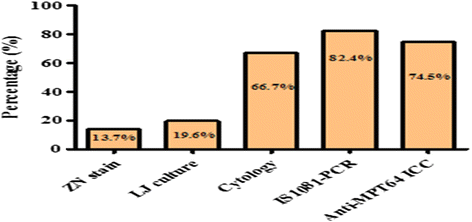

Supplement: Supplementary file 3 — Authors’ original file for figure 3 [file 12879_2014_585_MOESM3_ESM.gif]
